# Association of Gestation and Fetal Growth Restriction on Cardiovascular Health in Preterm-Born Children

**DOI:** 10.1016/j.jpeds.2022.09.057

**Published:** 2023-04

**Authors:** Christopher W. Course, Sarah J. Kotecha, Michael Cousins, Kylie Hart, John Lowe, W. John Watkins, Sailesh Kotecha

**Affiliations:** 1 Department of Child Health, Cardiff University School of Medicine, Cardiff, United Kingdom; 2 Department of Paediatrics, Cardiff and Vale University Health Board, Cardiff, United Kingdom

## Abstract

**Objectives:**

To prospectively evaluate the associations of early and current life factors, including gestational age and fetal growth restriction in preterm-born subjects, on cardiovascular health including measures of central and peripheral blood pressure and arterial stiffness and assess cardiovascular changes before and after acute exercise in preterm- and term-born school-aged children.

**Study design:**

From 240 children, aged 7-12 years, 204 (141 preterm-born and 63 term-born) had satisfactory data. An oscillometric device recorded cardiovascular measures before and after cycle ergometer exercise testing. Data were analyzed with multivariable linear regression and mediation.

**Results:**

Central systolic blood pressure (SBP) was 6.4 mmHg (95% CI, 1.2, 11.6) higher in preterm-born children with fetal growth restriction and 3.4 mmHg (0.02, 6.8) higher in those without fetal growth restriction when compared with term controls. Augmentation index was 4.1% (0.7, 7.4) higher in the preterm fetal growth restriction group when compared with those without fetal growth restriction but was similar between the latter group and term controls. Regression modelling showed gestational age, female sex, and antenatal smoking, but not fetal growth restriction, were significantly associated with SBP. In contrast, fetal growth restriction and fat mass index, but not gestation, were significantly associated with augmentation index. Cardiovascular exercise responses were similar between all 3 groups studied.

**Conclusions:**

Our data show the differential associations of prematurity and fetal growth restriction on central SBP and augmentation index. Cardiovascular responses to exercise were similar in all 3 groups. Preterm-born children with and without fetal growth restriction are at an increased risk of cardiovascular disease in adult life.

**Trial registration:**

URL: https://www.clinicaltrialsregister.eu/ctr-search/trial/2015-003712-20/GB: RHiNO, EudraCT: 2015-003712-20.

Preterm birth is increasingly recognized as resulting in adverse long-term cardiovascular outcomes.^1^ Peripheral arterial blood pressure has been shown to be increased in preterm-born subjects.^2^^,^^3^ Other important cardiovascular outcomes, especially those associated with endothelial function or arterial stiffness, are less well established for this population. We have previously shown increased systolic blood pressure (SBP) using the Avon Longitudinal Study of Parents and Children cohort especially in those born at 32 weeks or less gestation.^2^ However, markers for arterial stiffness and endothelial function including flow-mediated dilatation, distensibility coefficient, and pulse wave velocity (PWV) were not increased. In a review of the literature, there were suggestions that fetal growth restriction at birth may be associated with arterial stiffness^2^ but the independent contributions of prematurity and fetal growth restriction have been less studied. In contrast, an association between extremely preterm birth (<26 weeks' gestation) and increased augmentation index, an indirect measure of arterial stiffness based on aortic pulse wave reflection, has been reported at 11 years old in the EPICure cohort, which persisted at 19 years of age,^4^^,^^5^ but the investigators did not investigate the independent contributions of prematurity and fetal growth restriction.

In one study of 10 preterm-born young adults, SBP remained higher throughout exercise than in the 12 term-born controls.^6^ In another study of 13-year-old preterm-born children, prolonged recovery time for heart rate was noted after exercise when compared with term controls but blood pressure changes after exercise were not reported.^7^

We investigated cardiovascular outcomes including central and peripheral blood pressure measurements as well as augmentation index and PWV before and immediately after acute exercise in preterm-born children, with and without a history of fetal growth restriction, and a term-born control group. In addition, we studied which early life factors were associated with any significant outcomes.

## Methods

The Respiratory Health Outcomes in Neonates study (EudraCT: 2015-003712-20) has been described previously.^8^^,^^9^ As outlined in Figure 1 (available at www.jpeds.com), children from the previous Respiratory and Neurological Outcomes in children born Preterm study^10^ were supplemented with additional preterm-born (≤34 weeks' gestation) and term-born children (≥37 weeks' gestation), sourced from the NHS Wales Informatics Service, and were sent a respiratory and neurodevelopment questionnaire at 7-12 years of age. Responders were invited for a home or hospital visit to obtain anthropometric details and medical history (which was supplemented by examination of the child's medical records). Children with congenital malformations, significant cardiopulmonary disorder, neuromuscular disease or neurological impairment were excluded. Ethical approval was obtained from the South-West Bristol Research Ethics Committee (15/SW/0289). Parents gave informed written consent and children provided assent to participate. From 1122 (827 preterm-born, 295 term-born) responders, 767 were assessed at their homes and 240 from South Wales attended respiratory, cardiovascular, and exercise testing conducted by a trained research nurse and a trained pediatric research fellow at Children's Hospital for Wales, Cardiff, UK, between 2017 and 2019.

Following physical examination, the child's height was measured using a stadiometer (Seca 217, Seca Deutschland) and weighed using calibrated bioelectrical impedance floor scales (Tanita BC-420MA, Tanita Europe B.V.) from which their fat-mass index (FMI) and fat-free mass index indices were calculated. Fetal growth restriction was defined as birthweight <10th percentile adjusted for sex and gestation using LMS growth version 2.77 (Medical Research Council).

Vicorder (Smart Medical) was used to estimate hemodynamic measures and arterial stiffness measures including augmentation index, PWV and transit time^11^ during the trial's baseline assessment. This oscillometric method has been validated against applanation tonometry methods (such as SphygmoCor, AtCor Medical) in adult^12^ and pediatric studies,^13^ and has excellent intra- and interobserver repeatability. Internal software calculates values for central blood pressure by applying a previously described transfer function to brachial pulse waveforms.^11^ Pulse wave analysis identifies the first and second systolic pressure peaks, which reflect the systolic pressure resulting from ventricular ejection and that resulting from the reflected aortic pulse wave respectively, allowing derivation of augmentation pressure (difference between second and first systolic peak) and augmentation index (augmentation pressure expressed as a percentage of the central pulse pressure). By applying a proprietary algorithm to pulse wave analysis, estimates for stroke volume and cardiac output are generated. The child was placed in a supine position with the cuffs placed over the right brachial and femoral arteries. Following baseline measurements, the child underwent a protocolised cardiopulmonary exercise testing using a cycle ergometer (Lode) as previously described.^9^ Briefly, the child experienced minimally loaded peddling for 3 minutes after which the load was increased by 10 watts every minute as a ramp (1 W/6 seconds). Exercising continued until participants were unable to maintain a cadence of >60 rpm. A ‘maximal test’ fulfilled 2 of the following; reached 80% of their predicted maximal heart rate; reached peak oxygen consumption rate plateau; respiratory exchange ratio >1 or showing signs of volitional exhaustion (assessed by pictorial Omni scale^14^). Repeat measures were made using the Vicorder within 5 minutes of completing acute exercise.

Data are presented as mean, SD; 95% CI or number and percentage as appropriate. Data were compared using independent samples (or paired for exercise data) *t* test or one-way ANOVA with post hoc Bonferroni correction as appropriate. Pearson $\chi^{2}$ was used to analyze categorical data. Univariable and forward stepwise multivariable linear regression modelling were performed to identify associations with cardiovascular variables. Mediation analyses were performed with MPlus version 7.4 (Muthen & Muthen), and all other analyses were performed using SPSS version 26.0 (IBM). Results with SD z-score of greater than ±3.29 (representing the top and bottom 0.1% of the normal distribution) were excluded from analyses as they were considered implausible. *P*-value of < .05 was considered statistically significant.

## Results

From 240 children who participated, 219 had valid cardiovascular assessments (Figure 1). An additional 12 children with measurements >±3.29 SDs and 3 term-born children with fetal growth restriction were excluded resulting in 204 (141 preterm-born and 63 term-born) children. There were no significant differences for sex, ethnicity, or anthropometric measurements between the groups (Table I). The preterm-born children were 7 months older than term-born children at time of assessment. As anticipated, preterm-born children had lower gestational age, birthweight, and increased antenatal maternal smoking and morbidities associated with preterm birth when compared with the term-born controls.

**Table I:** Demographics of included children for baseline assessment

Variables	Preterm born (all) n = 141	Preterm-born fetal growth restriction n = 24	Preterm-born AGA n = 117	Term born n = 63
Current Status				
Sex (male), n(%)	69 (48.9%)	8 (33.3)	61 (52.1)	32 (50.8)
Ethnicity (white) n(%)	133 (94.3%)	22 (91.7)	111 (94.9)	62 (98.4)
Age at testing (y), mean (SD)	11.07 (1.23)^##^	11.01 (1.27)	11.08 (1.23)^‡‡^	10.49 (1.12)
Height (cm), mean (SD)	146.06 (10.29)	141.88 (11.38)	146.91 (9.89)	144.21 (9.67)
Height (z-score), mean (SD)	0.27 (1.04)	−0.35 (0.99)∗∗^††^	0.40 (1.01)	0.50 (1.01)
Weight (kg), mean (SD)	39.57 (10.62)	36.30 (10.61)	40.24 (10.55)	38.50 (10.78)
Weight (z-score), mean (SD)	0.31 (1.17)	−0.21 (1.29)∗^††^	0.41 (1.12)	0.51 (1.05)
Body mass index (z-score), mean (SD)	0.19 (1.31)	−0.10 (1.49)	0.24 (1.26)	0.37 (1.11)
FMI (kg/m^2^), mean (SD)	4.03 (2.21)	3.98 (2.08)	4.04 (2.25)	3.93 (2.20)
Fat free mass index (kg/m^2^), mean (SD)	14.27 (1.55)	13.77 (1.65)	14.37 (1.51)	14.31 (1.33)
Neonatal history				
Gestational age (wk), mean (SD)	30.76 (2.79)^###^	30.08 (2.33)^†††^	30.89 (2.86)^‡‡‡^	40.24 (1.18)
Birthweight (g), mean (SD)	1597 (578.0)^###^	997 (320.6)∗∗∗^†††^	1720 (541.1)^‡‡‡^	3579 (493.6)
Birthweight (z-score), mean (SD)	0.10 (1.28)	−1.86 (0.67)∗∗∗^†††^	0.50 (0.97)	0.17 (0.87)
Antenatal smoking, n (%)	15 (10.8%)^#^	3 (12.5)^†^	12 (10.3)^‡^	1 (1.6)
Chronic lung disease of prematurity, n (%)	42 (29.8%)^###^	6 (25.0)^†††^	36 (30.8)^‡‡‡^	0 (0)
Patent ductus arteriosus, n (%)	13 (9.4%)^#^	3 (12.5)^††^	10 (8.5)^‡^	0 (0)
Necrotizing enterocolitis, n (%)	8 (5.8%)	2 (8.3)^†^	6 (5.1)	0 (0)
Retinopathy of prematurity, n (%)	9 (6.4%)^#^	1 (4.2)	8 (6.8)^‡^	0 (0)
Intraventricular haemorrhage, n (%)	17 (12.1%)^##^	2 (8.3)^†^	15 (12.8)^‡‡^	0 (0)

Preterm-born vs Term-born: ^#^*P* < .05, ^##^*P* < .01, ^###^*P* < .001.

Preterm-born fetal growth restriction vs Preterm-born AGA: ∗*P* < .05, ∗∗*P* < .01, ∗∗∗*P* < .001.

Preterm-born fetal growth restriction vs Term-born: †*P* < .05, ††*P* < .01, †††*P* < .001.

Preterm-born AGA vs Term-born: ‡*P* < .05, ‡‡*P* < .01, ‡‡‡*P* < .001.

Antenatal smoking data missing for 2 preterm-born AGA subjects.

The preterm-born children had greater peripheral (mean 120.3; [SD: 9.7] mmHg vs 116.8 [9.3] mmHg, *P* = .017) and central SBP (112.2 [9.2] mmHg vs 108.3 [8.4] mmHg; *P* = .005) when compared with term-born children (Table II). In addition, significantly more of the preterm-born children had a peripheral SBP >90th percentile corrected for age, sex, and height^15^ (55.3% vs 36.5%; *P* = .013). Augmentation index and PWV were not different from the term-born controls. When preterm-born children who had fetal growth restriction at birth were compared with the term group, they had higher central SBP (mean difference of 6.4 mmHg; 95% CI, 1.2, 11.6; *P* = .01). Preterm-born children who were appropriate birth weight for gestational age (AGA) also had increased central SBP (3.4; 0.02, 6.8; *P* = .048) when compared with term-born children but the difference between the fetal growth restriction and AGA preterm groups was not significantly different (3.0; −1.8, 7.8; *P* = .4). The difference between central and peripheral SBP was lower in the preterm fetal growth restriction group when compared with the preterm AGA (−2.2; 95% CI, −4.3, −0.2; *P* = .029) and term-born (−2.2; −4.4,-0.02; *P* = .048) groups. This difference was largely due to the higher central SBP in preterm-born children with fetal growth restriction. A larger proportion of the preterm-born fetal growth restriction group had peripheral SBP >90th percentile when compared with term-born children (70.8% vs 36.5%; *P* = .012), but no significant difference was seen on comparison with the preterm-born AGA group (70.8% vs 52.1%; *P* = .275).

**Table II:** Effect of prematurity and fetal growth restriction on cardiovascular measures in childhood

Variables	Preterm (all) n = 141 Mean (SD)	Preterm fetal growth restriction n = 24 Mean (SD)	Preterm AGA n = 117 Mean (SD)	Term n = 63 Mean (SD)	Preterm vs term Mean difference [95% CI]	Preterm fetal growth restriction vs preterm AGA Mean difference [95% CI]	Preterm fetal growth restriction vs term Mean difference [95% CI]	Preterm AGA vs term Mean difference [95% CI]
Peripheral systolic BP (mmHg]	120.3 (9.7)	120.9 (11.7)	120.1 (9.3)	116.8 (9.3)	3.5† [0.6, 6.4]	0.8 [−4.4, 6.0]	4.2 [−1.4, 9.8]	3.4 [−0.3, 7.0]
Peripheral diastolic BP (mmHg]	57.5 (7.6)	59.8 (7.9)	57.0 (7.4)	55.7 (6.8)	1.8 [−0.4, 4.0]	2.7 [−1.2, 6.7]	4.0 [−0.2, 8.3]	1.3 [−1.4, 4.1]
Peripheral pulse pressure [mmHg]	62.7 (11.0)	61.2 (10.9)	63.1 (11.0)	61.0 (10.5)	1.7 [−1.5, 6.0]	−1.9 [−7.8, 4.0]	0.1 [−6.2, 6.4]	2.1 [−2.0, 6.1]
Central systolic BP [mmHg]	112.2 (9.2)	114.7 (10.8)	111.7 (8.8)	108.3 (8.4)	3.9† [1.2, 6.6]	3.0 [−1.8, 7.8]	6.4† [1.2, 11.6]	3.4∗ [0.02, 6.8]
Central diastolic BP [mmHg]	57.5 (7.5)	59.8 (7.9)	57.1 (7.4)	55.7 (6.8)	1.8 [−0.4, 4.0]	2.7 [−1.2, 6.6]	4.0 [−0.2, 8.3]	1.3 [−1.4, 4.1]
Central pulse pressure [mmHg]	54.7 (9.7)	55.0 (10.1)	54.7 (9.9)	52.6 (9.1)	2.1 [−0.8, 5.0]	0.3 [−4.9, 5.5]	2.4 [−3.2, 8.0]	2.1 [−1.6, 5.7]
Difference between peripheral and central systolic BP [mmHg]	8.1 (3.8)	6.2 (3.7)	8.4 (3.7)	8.4 (4.1)	−0.4 [−1.5, 0.8]	−2.2∗ [−4.3, −0.2]	−2.2∗ [−4.4, −0.02]	0.0 [−1.5, 1.4]
End systolic pressure [mmHg]	106.5 (11.4)	108.8 (11.5)	106.1 (11.3)	103.4 (11.0)	3.1 [−0.3, 6.5]	2.8 [−3.3, 8.9]	5.4 [−1.1, 12.0]	2.6 [−1.6, 6.9]
End systolic pressure index	0.9 (0.2)	0.9 (0.1)	0.9 (0.2)	0.9 (0.2)	0.0 [−0.1, 0.0]	0.0 [−0.1, 0.1]	0.0 [−0.1, 0.0]	0.0 [−0.1, 0.0]
Mean arterial pressure [mmHg]	83.3 (7.7)	86.4 (8.8)	82.6 (7.3)	80.8 (6.8)	2.5∗ [0.3, 4.7]	3.8 [−0.2, 7.8]	5.6† [1.4, 9.9]	1.8 [−1.0, 4.6]
Central augmentation pressure [mmHg]	6.9 (4.1)	8.6 (4.2)	6.5 (3.9)	6.0 (4.0)	0.9 [−0.3, 2.1]	2.1 [−0.1, 4.2]	2.6∗ [0.3, 4.9]	0.5 [−1.0, 2.0]
Central augmentation index [%]	12.1 (6.2)	15.5 (7.0)	11.4 (5.8)	11.1 (6.4)	1.0 [−0.8, 2.9]	4.1∗ [0.7, 7.4]	4.4∗ [0.9, 8.0]	0.4 [−2.0, 2.7]
Subendocardial viability ratio [%]	200.6 (77.0)	181.7 (68.5)	204.4 (78.3)	195.5 (68.4)	5.1 [−17.2, 27.3]	−22.7 [−62.9, 17.5]	−13.8 [−56.8, 29.2]	8.9 [−37.0, 19.1]
Pulse pressure index	1.1 (0.1)	1.2 (0.2)	1.1 (0.1)	1.1 (0.1)	0.00 [−0.04, 0.05]	0.1∗ [0.001, 0.2]	0.1 [−0.0, 0.2]	0.0 [−0.1, 0.0]
Transit time [m/s]	62.7 (8.9)	60.1 (9.4)	63.3 (8.7)	60.0 (8.0)	2.7∗ [0.1, 5.3]	−3.2 [−7.8, 1.4]	0.1 [−4.9, 5.0]	3.3∗ [0.03, 6.5]
Pulse wave velocity [m/s]	9.7 (1.4)	9.9 (1.6)	9.7 (1.4)	9.8 (1.5)	−0.1 [−0.5, 0.4]	0.3 [−0.5, 1.0]	0.1 [−0.7, 1.0]	−0.1 [−0.7, 0.4]
Stroke volume [ml]	98.8 (29.4)	99.5 (32.5)	98.7 (28.8)	102.9 (30.5)	−4.1 [−13.0, 4.8]	0.8 [−15.3, 16.9]	−3.5 [−20.7, 13.8]	−4.3 [−15.5, 7.0]
Cardiac output [L/min]	7.0 (2.4)	7.2 (2.3)	6.9 (2.4)	7.1 (2.2)	−0.1 [−0.8, 0.6]	0.3 [−1.0, 1.6]	0.1 [−1.3, 1.5]	−0.2 [−1.1, 0.7]
Cardiac index [L/min/m^2^]	5.6 (2.3)	6.2 (2.7)	5.5 (2.2)	5.9 (2.2)	−0.3 [−0.9, 0.4]	0.7 [−0.5, 1.9]	0.4 [−0.9, 1.6]	0.4 [−1.2, 0.5]
Heart rate [beats/min]	70.3 (11.7)	73.8 (13.1)	69.6 (11.3)	68.8 (8.7)	1.5 [−1.7, 4.8]	4.1 [−1.7, 10.0]	4.9 [−1.3, 11.2]	0.8 [−3.3, 4.9]

∗ *P* < .05.

† *P* < .01.

Preterm-born children with a history of fetal growth restriction, when compared with term-born children, had higher mean arterial pressure (86.4 [8.8] vs 80.8 [6.8] mmHg; *P* = .005), augmentation pressure (8.6 [4.2] vs 6.0 [4.0] mmHg; *P* = .021), and augmentation index (15.5% [7.0] vs 11.1 [6.4]; *P* = .009). Augmentation index was higher in the preterm-born fetal growth restriction children when compared with preterm-born AGA children (15.5 [7.0] vs 11.4 [5.8]; *P* = .011). No differences were noted for subendocardial viability ratio, PWV, stroke volume, and cardiac output or cardiac index between the preterm fetal growth restriction group when compared with the other 2 groups.

We next used linear regression analysis to identify potential predictors for central SBPs and augmentation index (Table III). Gestational age (β −0.32; *P* = .012), FMI (0.70; *P* = .015), female sex (3.65; *P* = .004), fetal growth restriction (4.27; *P* = .029), and antenatal maternal smoking (5.82; *P* = .013) were significantly associated with increased central SBP in univariable analysis. Gestational age (−0.26; *P* = .037), female sex (3.53; *P* = .005), and maternal antenatal smoking (5.19; *P* = .025), but not fetal growth restriction nor FMI, remained significantly associated with central SBP in multivariable regression. Fetal growth restriction (β 4.28; *P* = .001), body mass index (0.50; *P* = < .001), FMI (0.73; *P* = < .001) and fat-free mass index (0.93; *P* = .001), but not gestation, were significantly associated with augmentation index in univariable analysis. In the multivariable regression model, fetal growth restriction and FMI (or body mass index) remained significantly associated with augmentation index (*P* = < .001).

**Table III:** Linear regression analysis of associations of central SBP and augmentation index in all children. Forward stepwise linear regression used for multivariable models

Variables	Central SBP			Augmentation index		
	Univariable analyses			Univariable analyses		
	Beta	Standard Error	Significance	Beta	Standard Error	Significance
Gestational age (weeks)	−0.32	0.13	0.012∗	−0.12	0.09	0.18
Birthweight (z-score)	−0.46	0.55	0.41	−0.50	0.37	0.18
Height (z-score)	0.30	0.62	0.63	−0.40	0.42	0.34
Body mass index (z-score)	0.92	0.51	0.07	1.24	0.34	<0.001‡
FMI	0.70	0.29	0.015∗	0.73	0.19	<0.001‡
Fat Free Mass Index	0.65	0.43	0.42	0.93	0.29	0.001†
Sex (ref = Male)	3.65	1.26	0.004†	0.44	0.87	0.61
Fetal growth restriction (ref = No)	4.27	1.96	0.029∗	4.28	1.31	0.001†
Antenatal smoking (ref = No)	5.82	2.34	0.013∗	0.83	1.61	0.60

	Multivariable analysis of central SBP		
	Beta	Standard Error	Significance
Gestational age	−0.26	0.12	0.037∗
Sex (ref = Male)	3.53	1.24	0.005†
Antenatal smoking (ref = No)	5.19	2.31	0.025∗

	Multivariable analysis of augmentation Index		
	Beta	Standard Error	Significance
Fat mass index	0.73	0.18	<0.001‡
Fetal growth restriction (ref = No)	4.29	1.26	0.001†

∗ *P* < .05.

† *P* < .01.

‡ *P* < .00.

Next, we used mediation analyses to investigate the contributions of early life factors to increased central SBP and augmentation index (Figure 2; available at www.jpeds.com). Gestational age and sex had a direct effect on increased central SBP, but fetal growth restriction was consequent of preterm birth and did not appear to directly affect increased central SBP. Augmentation index was affected by fetal growth restriction, but not gestational age nor sex.

**Figure 3:**
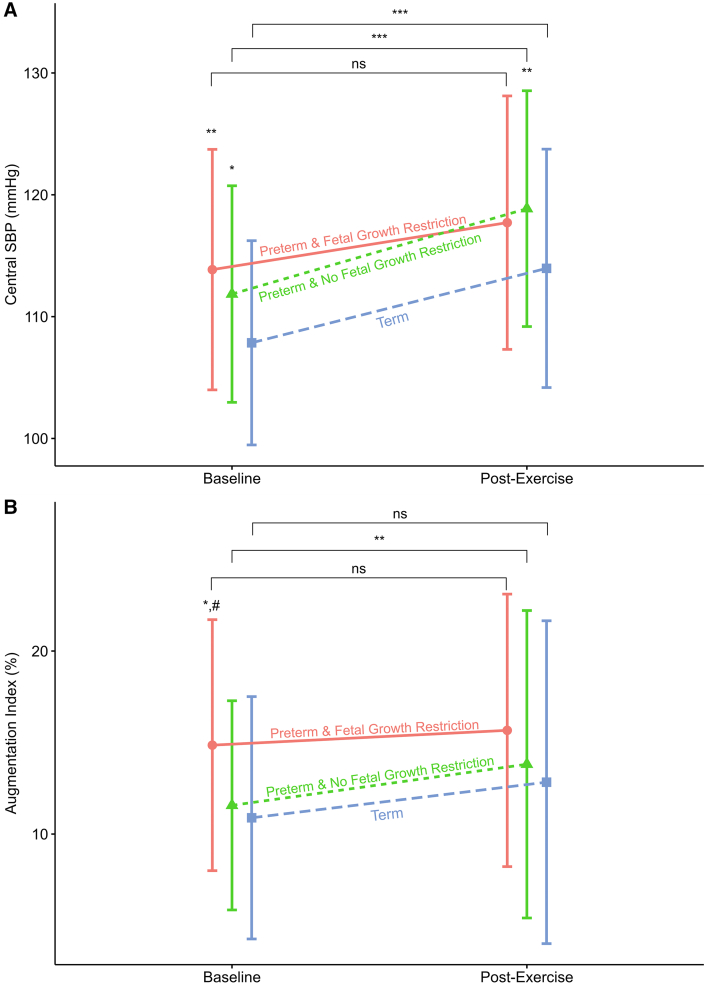
**A,** Central SBP and **B,** augmentation index by group at baseline and after cardiopulmonary exercise testing (n = 179). Groups labelled with text and shapes (Circle = Preterm and fetal growth restriction, Triangle = Preterm & No fetal growth restriction, Square = Term). Shape also represent respective group mean, SD given by vertical bars. At baseline and post cardiopulmonary exercise testing time points ∗*P* < .05 compared with term, ∗∗*P* < .01 compared with term, #*P* < .05 compared with preterm AGA. At baseline, for central SBP both preterm-born groups had significantly higher measurements than term-born. Preterm-born fetal growth restriction group significantly higher augmentation index than preterm-born AGA and term-born children. Post cardiopulmonary exercise testing Preterm-born AGA had significantly higher SBP than term-born. No differences seen in augmentation index post cardiopulmonary exercise testing. Horizontal brackets compare baseline with post cardiopulmonary exercise testing: ∗*P* < .05, ∗∗*P* < .01, ∗∗∗*P* < .001. Preterm-born AGA and Term-born showed significant increase in their respective mean SBP between baseline and post cardiopulmonary exercise testing. Only Preterm-born AGA showed a significant increase in augmentation index between baseline and post cardiopulmonary exercise testing.

**Figure 1:**
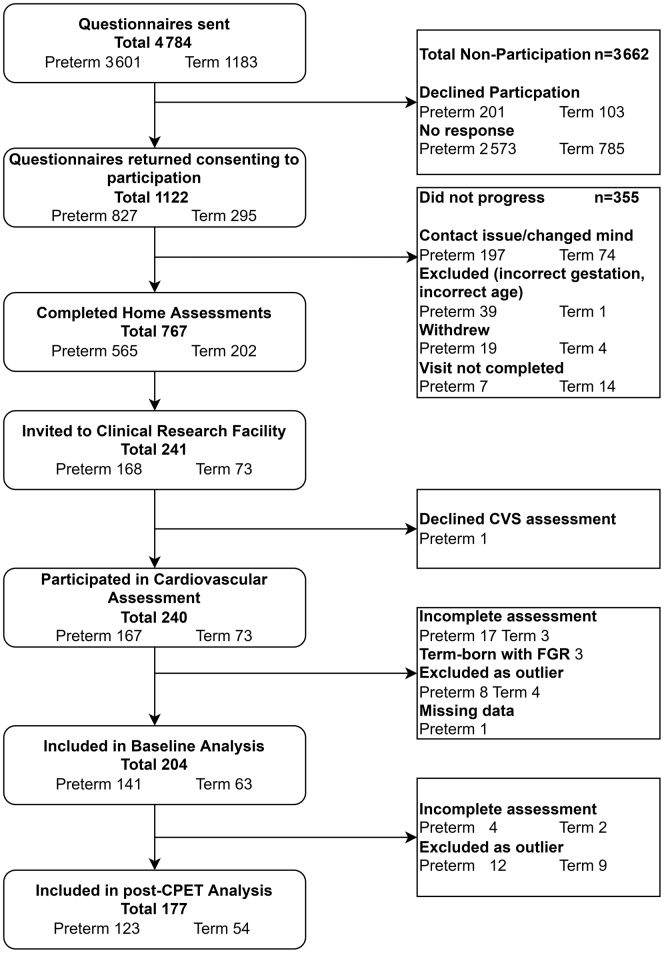
Flow diagram showing recruitment to Respiratory Health Outcomes in Neonates Study (RHiNO) Study and the cardiovascular assessment stage of the study.

**Figure 2:**
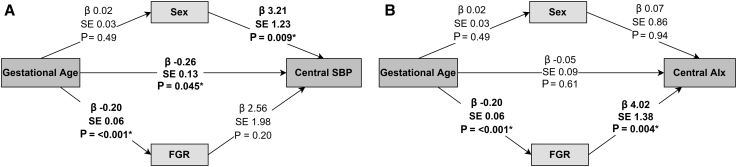
Results of mediation analysis for **A,** Central SBP and **B,** augmentation index baseline data. Beta (β), standard error (SE) and *P* value given for interactions between variables, with arrows showing direction of relationships. *FGR*, fetal growth restriction.

We assessed the effects of cardiopulmonary exercise testing on cardiovascular measurements. From 216 with baseline data, 211 (98%) underwent cardiopulmonary exercise testing and satisfactory data were available for 177 (82%, 123 preterm-born and 54 term-born) children after quality control and outliers were removed (demographics shown in Table IV; available at www.jpeds.com). In general, the peripheral SBP, central SBP, central diastolic blood pressure, and mean arterial pressure increased in the preterm-born children with and without fetal growth restriction when compared with term-born children after completing cardiopulmonary exercise testing (Tables V and VI; available at www.jpeds.com. Figure 3). As at baseline, measurements remained higher in the preterm groups than their term counterparts. There were no postexercise differences in augmentation index or other markers of arterial stiffness between the 3 groups. The preterm-born AGA group increased their augmentation index after cardiopulmonary exercise testing (2.3%; 95% CI, 0.6%, 3.9%; *P* = .008) but a similar increase was not observed in the preterm-born fetal growth restriction and term-born groups.

## Discussion

The findings show that prematurity and fetal growth restriction in the preterm-born group act differently on the developing cardiovascular system. Several studies, including metanalyses,^3^^,^^16^ have demonstrated that prematurity is associated with increased SBP in young adult life. The effects of prematurity and fetal growth restriction on later cardiovascular health in the existing literature are not clear, possibly due to small sample sizes available. Our data show that central SBP is associated with prematurity and not fetal growth restriction; in contrast, augmentation index is more associated with fetal growth restriction in the preterm population and less so with prematurity alone. The degree of increased SBP and sex-related differences we observed are in keeping with previously published studies and meta-analyses.^2^^,^^3^^,^^16^ Whilst these differences appear low in childhood, because SBP tracks throughout life, these differences are likely to increase with age.^17^ In adults, central SBP is better related to future cardiovascular events than peripheral measurements,^18^ and each 20 mmHg increase is associated with a two-fold increased risk of cardiovascular mortality,^19^ hence we focused more on central SBP. Antenatal maternal smoking has also been shown to be associated with increases in SBP in adolescence^20^; and increased body mass index and adiposity in childhood have also been shown to be associated with increased SBP and adverse adult cardiovascular outcomes.^21^ However, the majority of children included in these studies were term-born. In contrast, Flahault et al did not demonstrate an association between adiposity and increased SBP in preterm-born subjects.^22^ In our study, FMI was significantly associated with central SBP in univariable regression modeling but did not reach significance in multivariable modeling. Our data showed an association between fetal growth restriction and central SBP in univariable regression analyses but was no longer significant in multivariable modeling. We excluded 3 term-born children with fetal growth restriction to avoid influencing the effects of fetal growth restriction associated with preterm-birth on cardiovascular outcomes. Meta-analysis has shown that adults born with low birth weight (<2500 g) have an increased SBP,^23^ and the recent UK Biobank data showed that adults with low birthweight are at increased risk of cardiovascular disease,^24^ but neither study assessed the separate effects of gestational age and birthweight on cardiovascular outcomes. Preterm-born children are known to be at increased risk of sleep-disordered breathing which can impact autonomic cardiovascular control^25^; however, none of the children in our cohort were under the care of sleep disorder services.

We did, however, find a relationship between fetal growth restriction and augmentation index which appeared to be independent of gestation. Increased augmentation index reflects increased arterial stiffness and premature vascular ageing. Adult studies have shown that an increase in augmentation index of approximately 4% increases the risk of early coronary artery disease^26^ highlighting the importance of identifying these individuals early in life. In our population the augmentation index difference was >4% when the preterm population with fetal growth restriction were compared with those without fetal growth restriction and term controls. This is an important finding that may be associated with the longer-term atherosclerotic morbidity in preterm-born adults as recently reported by Crump et al.^27^ The existing literature on the relationship between preterm-birth and augmentation index is conflicting. The EPICure study did not report any differences in peripheral or central SBP at 11 years of age in extremely preterm-born survivors (<26 weeks' gestation at birth) but reported 5% increase in augmentation index,^4^ which persisted to 19 years of age.^5^ A study of British young adults born preterm in the 1980s noted a reduction in aortic lumen size, also associated with an increased augmentation index of approximately 10%, in the preterm-born group^28^; however, other studies have not noted a relationship between augmentation index and preterm-birth.^29^ How the findings from these studies related to fetal growth restriction were not investigated. An Australian study of 71 term- and preterm-born young adolescents with and without histories of fetal growth restriction^30^ found that fetal growth restriction was a stronger predictor of SBP than prematurity, but that the combination of prematurity and fetal growth restriction (n = 14) had a larger effect on augmentation index; a difference of 9.7% was noted between the preterm growth restricted group compared with preterm control, growth restricted term and term control populations. Using a comparatively larger population of children, our study showed that augmentation index was significantly associated with fetal growth restriction but not with prematurity in multivariable regression and mediation models. Our mediation model has demonstrated that the previously described relationship between preterm-birth and elevated augmentation index may be mediated by fetal growth restriction.

Data for cardiovascular changes after exercise in preterm-born subjects are limited. One recent study showed that preterm-born individuals have smaller left ventricular volumes at baseline in adolescents, with a history of fetal growth restriction being associated with a reduced left ventricular output.^31^ A Spanish study also showed reduced left ventricular size and reduced cardiac efficiency in term- and preterm-born children with severe fetal growth restriction.^32^ This could potentially limit cardiovascular exercise tolerance. We noted that preterm- and term-born children appeared to have similar increases for peripheral and central SBP after exercise, with preterm-born children remaining generally higher than the term population as at baseline. No significant differences were noted after exercise for central diastolic blood pressure and mean arterial pressure compared with term-born children post-exercise. Minimal increases were noted for augmentation index, with the baseline relationships of highest values in the preterm group with fetal growth restriction and lowest in the term controls remaining. Although limitations in exercise have been noted for preterm-born children, these are more likely to be due to respiratory dysfunction associated with prematurity,^33^^,^^34^ rather than cardiovascular responses, at least in childhood.

These data suggest that long term cardiovascular assessment, including measurement of blood pressure as a minimum screening tool, is essential to prevent longer term morbidity and mortality associated with preterm birth.

## Data Statement

Data sharing statement available at www.jpeds.com.

## Figures and Tables

**Table IV:** Demographics for post cardiopulmonary exercise testing assessment

	Preterm born (all) n = 123	Preterm born fetal growth restriction n = 21	Preterm born AGA n = 102	Term born n = 54
Current Status				
Sex (male), n(%)	60 (48.8)	6 (28.6)∗	54 (52.9)	25 (46.3)
Ethnicity (white), n(%)	115 (93)	19 (90.5)	96 (94.1)	53 (98)
Age at testing (y), mean (SD)	11.1 (1.2)^##^	11.0 (1.3)	11.1 (1.3)^‡^	10.5 (1.1)
Height (cm), mean (SD)	146.1 (10.4)	142.0 (12.1)	146.9 (9.9)	144.5 (9.8)
Height (z-score), mean (SD)	0.3 (1.0)	−0.32 (1.06)∗^††^	0.38 (0.94)	0.51 (1.02)
Weight (kg), mean (SD)	39.8 (10.8)	36.2 (10.9)	40.6 (10.7)	39.0 (11.1)
Weight (z-score), mean (SD)	0.3 (1.1)	−0.21 (1.30)∗^†^	0.45 (1.08)	0.6 (1.0)
Mass index (z-score), mean (SD)	0.22 (1.31)	−0.13 (1.50)	0.30 (1.26)	0.42 (1.11)
FMI (kg/m^2^), mean (SD)	4.1 (2.2)	4.01 (2.04)	4.11 (2.30)	4.06 (2.26)
Fat free mass index (kg/m^2^), mean (SD)	14.3 (1.6)	13.67 (1.65)	14.45 (1.56)	14.40 (1.36)
Neonatal history				
Gestational age (wk), mean (SD)	30.5 (2.8)^###^	29.67 (2.33)^†††^	30.65 (2.89)^‡‡‡^	40.00 (1.15)
Birthweight (g), mean (SD)	1593 (576)^###^	987 (333.0)∗∗∗^†††^	1718 (535.4)^‡‡‡^	3537 (484.8)
Birthweight (z-score), mean (SD)	0.1 (1.3)	−1.83 (0.69)∗∗∗^†††^	0.50 (0.98)^‡^	0.12 (0.89)
Antenatal smoking, n(%)	12 (10)	3 (14.3)^†^	9 (8.8)	1 (2.0)
Chronic lung disease of prematurity, n (%)	34 (28)^###^	5 (23.8)^†††^	29 (28.4)^‡‡‡^	0 (0)
Patent ductus arteriosus, n (%)	11 (9)^#^	2 (9.5)^†^	9 (8.8)^‡‡^	0 (0)
Necrotizing enterocolitis, n (%)	7 (6)	1 (4.8)	6 (5.9)	0 (0)
Retinopathy of prematurity, n (%)	8 (7)	1 (4.8)	7 (6.9)^‡^	0 (0)
Intraventricular haemorrhage, n (%)	16 (13)^##^	2 (9.5)^†^	14 (13.8)^‡‡^	0 (0)

Preterm-born vs Term-born: ^#^*P* < .05, ^##^*P* < .01, ^###^*P* < .001.

Preterm-born fetal growth restriction vs Preterm-born AGA: ∗*P* < .05, ∗∗*P* < .01, ∗∗∗*P* < .001.

Preterm-born fetal growth restriction vs Term-born: †*P* < .05, ††*P* < .01, †††*P* < .001.

Preterm-born AGA vs Term-born: ‡*P* < .05, ‡‡*P* < .01, ‡‡‡*P* < .001.

Antenatal smoking data missing for 2 preterm-born AGA subjects.

**Table V:** Pre/post cardiopulmonary exercise testing data for preterm-born vs term-born children

	Preterm born children (n = 123)			Term born children (n = 54)			Preterm vs term	
	Baseline Mean (SD)	Post cardiopulmonary exercise testing Mean (SD)	Mean difference (SD) [95% CI]	Baseline Mean (SD)	Post cardiopulmonary exercise testing Mean (SD)	Mean difference (SD) [95% CI]	Mean difference Post cardiopulmonary exercise testing [95% CI]	Mean difference Pre vs post cardiopulmonary exercise testing[95% CI]
Peripheral SBP [mmHg]	120.3 (9.8)	125.8 (10.9)	5.5‡ (11.2) [3.5, 7.5]	116.3 (9.2)	122.0 (10.3)	5.7† (11.5) [2.5, 8.8]	3.8∗ [0.3, 7.2]	−0.2 [−3.8, 3.5]
Peripheral diastolic BP [mmHg]	57.3 (7.7)	61.5 (9.6)	4.2‡ (9.4) [2.5, 5.8]	56.1 (6.8)	58.4 (9.3)	2.3 (10.7) [−0.6, 5.2]	3.1 [−0.1, 6.1]	1.8 [−1.3, 5.0]
Peripheral pulse pressure [mmHg]	63.0 (11.2)	64.3 (13.9)	1.4 (14.7) [−1.3, 4.0]	60.2 (10.2)	63.3 (11.4)	3.1 (11.5) [−0.0, 6.2]	1.0 [−3.2, 5.3]	−1.8 [−6.2, 2.7]
Central SBP [mmHg]	112.2 (9.1)	118.7 (9.8)	6.5‡ (9.5) [4.8, 8.2]	107.9 (8.4)	114.0 (9.8)	6.1‡ (10.6) [3.2, 9.0]	4.7† [1.6, 7.9]	0.4 [−2.8, 3.5]
Central diastolic BP [mmHg]	57.3 (7.7)	61.7 (9.2)	4.4‡ (9.5) [2.7, 6.1]	56.1 (6.8)	58.4 (9.3)	2.3 (10.7) [−0.6, 5.2]	3.3∗ [0.3, 6.3]	2.1 [−1.1, 5.3]
Central pulse pressure [mmHg]	54.9 (10.0)	57.2 (12.7)	2.3 (13.3) [−0.1, 4.7]	51.7 (8.6)	55.4 (10.5)	3.6† (9.8) [1.0, 6.3]	1.9 [−2.0, 5.7]	−1.3 [−5.3, 2.7]
Central augmentation pressure [mmHg]	6.9 (3.9)	8.3 (5.6)	1.5† (5.5) [0.5, 2.4]	5.8 (3.9)	7.3 (5.5)	1.6∗ (5.3) [0.1, 3.0]	1.0 [−0.8, 2.8]	−0.1 [−1.9, 1.6]
Central augmentation index [%]	12.1 (6.0)	14.1 (8.3)	2.0† (8.2) [0.5, 3.5]	10.9 (6.6)	12.8 (8.8)	1.9 (8.7) [−0.4, 4.3]	1.3 [−1.4, 4.0]	0.1 [−2.6, 2.8]
Mean arterial pressure [mmHg]	83.0 (7.6)	90.4 (8.0)	7.4‡ (7.8) [6.0, 8.8]	80.8 (7.1)	87.4 (8.2)	6.6‡ (9.8) [4.0, 9.3]	3.0∗ [0.4, 5.6]	0.7 [−2.0, 3.5]
Subendocardial viability ratio [%]	200.7 (78.1)	116.0 (25.6)	−84.**7**‡ (76.7) [−98.4, −71.0]	197.2 (68.7)	122.5 (30.2)	−74.7‡ (68.2) [−93.3, −56.1]	−6.5 [−15.2, 2.2]	−10.0 [−33.9, 13.9]
End systolic pressure [mmHg]	106.4 (11.3)	105.0 (10.7)	−1.3 (10.7) [−3.2, 0.6]	103.0 (11.1)	103.6 (9.9)	0.6 (11.9) [−2.6, 3.9]	1.4 [−2.0, 4.8]	−1.9 [−5.5, 1.6]
End systolic pressure index	0.9 (0.2)	0.8 (0.2)	−0.1‡ (0.1) [−3.2, 0.6]	0.9 (0.1)	0.8 (0.1)	−0.1‡ (0.2) [−0.2, -0.1]	−0.05∗ [−0.1, −0.02]	−0.03 [−0.08, 0.02]
Stroke volume [ml]	98.4 (29.0)	98.5 (27.0)	0.1 (33.1) [−5.8, 6.0]	99.2 (27.4)	100.9 (26.3)	1.8 (24.0) [−4.8, 8.3]	−2.4 [−11.0, 6.3]	−1.6 [−11.5, 8.2]
Cardiac output [L/min]	6.9 (2.4)	9.7 (2.9)	2.8‡ (3.1) [2.3, 3.4]	6.8 (2.1)	9.7 (2.7)	2.8‡ (2.2) [2.2, 3.4]	0.1 [−0.8, 1.0]	0.0 [−0.8, 0.8]
Cardiac index [L/min/m^2^]	5.5 (2.3)	7.8 (2.7)	2.3‡ (2.6) [1.8, 2.8]	5.6 (2.2)	7.9 (2.7)	2.3‡ (1.9) [1.8, 2.8]	−0.1 [−1.0, 0.7]	0.0 [−0.8, 0.8]

∗ *P* < .05.

† *P* < .01.

‡ *P* < .001.

**Table VI:** Pre/Post cardiopulmonary exercise testing data for preterm-born children with and without a history of fetal growth restriction

	Preterm born with fetal growth restriction (n = 21)			Preterm born AGA (n = 102)			AGA vs fetal growth restriction	
	Baseline Mean (SD)	Post cardiopulmonary exercise testing Mean (SD)	Mean difference ± SD [95% CI]	Baseline Mean (SD)	Post cardiopulmonary exercise testing Mean (SD)	Mean difference ± SD [95% CI]	Mean difference Post cardiopulmonary exercise testing [95% CI]	Mean difference Pre vs post cardiopulmonary exercise testing[95% CI]
Peripheral SBP [mmHg]	120.4 (11.0)	123.6 (11.6)	3.2 (11.7) [−2.1, 8.6]	120.3 (9.6)	126.3 (10.7)	6.0‡ (11.1) [3.8, 8.2]	2.6 [−2.5, 7.8]	2.8 [−2.6, 8.1]
Peripheral diastolic BP [mmHg]	59.8 (8.3)	62.6 (8.6)	2.9 (7.9) [−0.8, 6.5]	56.8 (7.5)	61.2 (9.9)	4.4‡ (9.6) [2.5, 6.3]	−1.5 [−6.0, 3.1]	1.6 [−2.9, 6.0]
Peripheral pulse pressure [mmHg]	60.6 (10.7)	60.5 (9.8)	−0.1 (11.9) [−5.5, 5.5]	63.5 (11.3)	65.1 (14.5)	1.6 (15.3) [−1.4, 4.6]	4.6 [−2.0, 11.2]	1.7 [−5.3, 8.7]
Central SBP [mmHg]	113.9 (9.9)	117.7 (10.4)	3.9 (9.7) [−0.5, 8.3]	111.9 (8.9)	118.9 (9.7)	7.0‡ (9.5) [5.2, 8.9]	1.2 [−3.5, 5.8]	3.2 [−1.4, 7.7]
Central diastolic BP [mmHg]	59.8 (8.3)	62.7 (8.6)	2.9 (7.9) [−0.8, 6.5]	56.8 (7.5)	61.5 (9.3)	4.7‡ (9.8) [2.8, 6.7]	−1.1 [−5.5, 3.2]	1.9 [−2.6, 6.4]
Central pulse Pressure [mmHg]	54.1 (9.8)	55.1 (9.8)	1.0 (11.1) [−4.1, 6.1]	55.1 (10.0)	57.7 (13.2)	2.6 (13.8) [−0.1, 5.3]	2.6 [−-3.4, 8.6]	1.6 [−4.8, 7.9]
Central augmentation pressure [mmHg]	8.1 (3.9)	8.8 (4.9)	0.8 (4.3) [−1.2, 2.7]	6.6 (3.9)	8.23 (5.8)	1.6† (5.8) [0.5, 2.7]	−0.6 [−3.3, 2.1]	0.8 [−1.8, 3.5]
Central augmentation index [%]	14.9 (6.9)	15.7 (7.5)	0.8 (7.2) [−2.5, 4.1]	11.6 (5.7)	13.8 (8.4)	2.3† (8.4) [0.6, 3.9]	−1.9 [−5.8, 2.1]	1.4 [−2.5, 5.3]
Mean arterial pressure [mmHg]	86.1 (8.5)	90.8 (8.3)	4.6† (6.5) [1.7, 7.6]	82.4 (7.2)	90.3 (8.0)	8.0‡ (7.9) [6.4, 9.5]	−0.4 [−4.3, 3.4]	3.3 [−0.3, 7.0]
Subendocardial viability ratio [%]	182.1 (73.0)	119.1 (20.2)	−63.0† (76.7) [−97.9, −28.0]	204.5 (78.9)	115.3 (26.6)	−89.2‡ (76.3) [−104.2, −74.2]	−3.8 [−16.0, 8.4]	−26.2 [−62.5, 10.0]
End systolic pressure [mmHg]	107.9 (10.6)	106.4 (9.5)	−1.5 (10.8) [−6.5, 3.4]	106.1 (11.4)	104.8 (11.0)	−1.3 (10.7) [−3.4, 0.8]	−1.6 [−6.7, 3.5]	0.2 [−4.9, 5.3]
End systolic pressure index	0.9 (0.2)	0.8 (0.1)	−0.1† (0.1) [−0.2, −0.0]	0.9 (0.2)	0.8 (0.2)	−0.1‡ (0.1) [−0.2, −0.1]	−0.1 [−0.1, 0.0]	−0.1 [−0.1, 0.0]
Stroke volume [ml]	96.1 (30.8)	92.1 (22.7)	−4.1 (33.1) [−19.2, 11.0]	98.9 (28.8)	99.9 (27.7)	1.0 (33.2) [−5.5, 7.5]	7.8 [−5.0, 20.6]	5.1 [−10.7, 20.8]
Cardiac output [L/min]	7.1 (2.3)	8.9 (2.0)	1.8∗ (3.0) [0.5, 3.2]	6.9 (2.5)	9.9 (3.0)	3.0‡ (3.1) [2.4, 3.7]	1.0 [−0.4, 2.4]	1.2 [−0.2, 2.7]
Cardiac index [L/min/m^2^]	6.2 (2.9)	7.6 (1.8)	1.4∗ (2.8) [0.1, 2.7]	5.4 (2.1)	7.9 (2.8)	2.5‡ (2.6) [2.0, 3.0]	0.3 [−1.0, 1.6]	1.1 [−0.2, 2.3]

∗ *P* < .05.

† *P* < .01.

‡ *P* < .001.

## Appendix

STROBE_checklist

Data Statement
