# Supplementary material for: Association of Gestation and Fetal Growth Restriction on Cardiovascular Health in Preterm-Born Children
Source: J Pediatr. 2023 Apr;255:42–9. doi: 10.1016/j.jpeds.2022.09.057 (PMC7614853; doi:10.1016/j.jpeds.2022.09.057)
Supplement: STROBE_checklist [file mmc1.doc]

STROBE Statement—checklist of items that should be included in reports of observational studies

|  | | Item No | Recommendation |
| --- | --- | --- | --- |
| **Title and abstract** | | 1 | (*a*) Indicate the study’s design with a commonly used term in the title or the abstract  *The study design is indicated in the abstract on page 4* |
|  |  |  | (*b*) Provide in the abstract an informative and balanced summary of what was done and what was found  *A balanced summary is provided in the abstract on page 4* |
| Introduction | | | |
| Background/rationale | | 2 | Explain the scientific background and rationale for the investigation being reported  *The scientific background rationale is explained in the introduction on page 5* |
| Objectives | | 3 | State specific objectives, including any prespecified hypotheses  *The objectives are specified in the final paragraph of the introduction on page 5-6.* |
| Methods | | | |
| Study design | | 4 | Present key elements of study design early in the paper  *The key elements of the study design are presented early in the paper in the methods section on page 6 and 7.* |
| Setting | | 5 | Describe the setting, locations, and relevant dates, including periods of recruitment, exposure, follow-up, and data collection  *The setting, location and relevant dates are presented in the methods section on page 6.* |
| Participants | | 6 | (*a*) *Cohort study*—Give the eligibility criteria, and the sources and methods of selection of participants. Describe methods of follow-up  *Details are given in page 6 of the manuscript in the methods section, and in Figure 1; online*  *Case-control study*—Give the eligibility criteria, and the sources and methods of case ascertainment and control selection. Give the rationale for the choice of cases and controls  *Cross-sectional study*—Give the eligibility criteria, and the sources and methods of selection of participants |
|  |  |  | (*b*) *Cohort study*—For matched studies, give matching criteria and number of exposed and unexposed  *Details are given in the first paragraph of the results section starting on page 8 and in Figure 1; online*  *Case-control study*—For matched studies, give matching criteria and the number of controls per case |
| Variables | | 7 | Clearly define all outcomes, exposures, predictors, potential confounders, and effect modifiers. Give diagnostic criteria, if applicable  *All variables are clearly defined in the methods section on pages 6, 7 and 8.* |
| Data sources/ measurement | | 8* | For each variable of interest, give sources of data and details of methods of assessment (measurement). Describe comparability of assessment methods if there is more than one group  *Clearly defined in the methods section on pages 6, 7 and 8.* |
| Bias | | 9 | Describe any efforts to address potential sources of bias  *Clearly defined in the methods section on pages 6, 7 and 8.* |
| Study size | | 10 | Explain how the study size was arrived at  *Study size is described in the methods on page 6 and results section starting on page 8* |
| Quantitative variables | | 11 | Explain how quantitative variables were handled in the analyses. If applicable, describe which groupings were chosen and why  *Statistical methods are given in the methods section on page 8.* |
| Statistical methods | | 12 | (*a*) Describe all statistical methods, including those used to control for confounding |
|  |  |  | (*b*) Describe any methods used to examine subgroups and interactions |
|  |  |  | (*c*) Explain how missing data were addressed |
|  |  |  | (*d*) *Cohort study*—If applicable, explain how loss to follow-up was addressed  *Case-control study*—If applicable, explain how matching of cases and controls was addressed  *Cross-sectional study*—If applicable, describe analytical methods taking account of sampling strategy |
|  |  |  | (*e*) Describe any sensitivity analyses  *Statistical methods are given in the methods section on page 8.* |
| Results | | | |
| Participants | 13* | (a) Report numbers of individuals at each stage of study—eg numbers potentially eligible, examined for eligibility, confirmed eligible, included in the study, completing follow-up, and analysed  *Numbers are reported in the first paragraph of the results section on page 8 and in Figure 1; online* | |
|  |  | (b) Give reasons for non-participation at each stage  *Reasons are given in the first paragraph of the results section on page 8and in Figure 1; online* | |
|  |  | (c) Consider use of a flow diagram | |
| Descriptive data | 14* | (a) Give characteristics of study participants (eg demographic, clinical, social) and information on exposures and potential confounders  *Characteristics are given in table 1 in the main paper and are discussed in paragraph 1 of the results section on page 8* | |
|  |  | (b) Indicate number of participants with missing data for each variable of interest  *Missing data is clearly reported in table 1 in the main paper.* | |
|  |  | (c) *Cohort study*—Summarise follow-up time (eg, average and total amount)  *Not applicable as all outcome variables measured at same point of time* | |
| Outcome data | 15* | *Cohort study*—Report numbers of outcome events or summary measures over time  *Outcomes are clearly reported in the results section on pages 8-11, and in tables 1-3 and figure 3 in the main paper, and tables 4-6; online and Figure 2; online.* | |
|  |  | *Case-control study—*Report numbers in each exposure category, or summary measures of exposure | |
|  |  | *Cross-sectional study—*Report numbers of outcome events or summary measures | |
| Main results | 16 | (*a*) Give unadjusted estimates and, if applicable, confounder-adjusted estimates and their precision (eg, 95% confidence interval). Make clear which confounders were adjusted for and why they were included  *Main results are given in in tables 1-3 and figure 3 in the main paper, and tables 4-6; online and Figure 2; online.. Standard deviation/error and 95% CIs are reported as appropriate* | |
|  |  | (*b*) Report category boundaries when continuous variables were categorized  *Boundaries are provided* | |
|  |  | (*c*) If relevant, consider translating estimates of relative risk into absolute risk for a meaningful time period  *Not applicable* | |
| Other analyses | 17 | Report other analyses done—eg analyses of subgroups and interactions, and sensitivity analyses  *All analyses are clearly reported in the results and the tables* | |
| Discussion | | | |
| Key results | 18 | Summarise key results with reference to study objectives  *Key results are summarised in the first paragraph of the discussion on page 11* | |
| Limitations | 19 | Discuss limitations of the study, taking into account sources of potential bias or imprecision. Discuss both direction and magnitude of any potential bias  *Limitations are discussed in the discussion on page 14.* | |
| Interpretation | 20 | Give a cautious overall interpretation of results considering objectives, limitations, multiplicity of analyses, results from similar studies, and other relevant evidence  *This is provided in the discussion on page 14.* | |
| Generalisability | 21 | Discuss the generalisability (external validity) of the study results  *This is provided in the discussion on page 14.* | |
| Other information | | | |
| Funding | 22 | Give the source of funding and the role of the funders for the present study and, if applicable, for the original study on which the present article is based  *Funding information is provided on page 2.* | |

*Give information separately for cases and controls in case-control studies and, if applicable, for exposed and unexposed groups in cohort and cross-sectional studies.

**Note:** An Explanation and Elaboration article discusses each checklist item and gives methodological background and published examples of transparent reporting. The STROBE checklist is best used in conjunction with this article (freely available on the Web sites of PLoS Medicine at http://www.plosmedicine.org/, Annals of Internal Medicine at http://www.annals.org/, and Epidemiology at http://www.epidem.com/). Information on the STROBE Initiative is available at www.strobe-statement.org.
