# Supplementary material for: Association of Gestation and Fetal Growth Restriction on Cardiovascular Health in Preterm-Born Children
Source: J Pediatr. 2023 Apr;255:42–9. doi: 10.1016/j.jpeds.2022.09.057 (PMC7614853; doi:10.1016/j.jpeds.2022.09.057)
Supplement: Data Statement [file mmc2.docx]

Data Sharing Statement

Data from the RHiNO study is available to research collaborators subject to confidentiality and non-disclousre agreements. Contact Professor Sailesh Kotecha (kotechas@cardiff.ac.uk) for any data requests.
